# Analysis of Cytological Markers of Cellular Senescence in High‐Risk Cardiovascular Patients After COVID‐19

**DOI:** 10.1155/bmri/5553698

**Published:** 2026-08-05

**Authors:** Vitalii Kyryk, Alina Ustymenko, Oleg Tsupykov, Oleksandr Skarzhevskyi, Oleksandr Parkhomenko

**Affiliations:** ^1^ Institute of Genetic and Regenerative Medicine, Strazhesko National Scientific Center of the National Academy of Medical Sciences of Ukraine, Kyiv, Ukraine; ^2^ Laboratory of Pathophysiology and Immunology, Chebotarev State Institute of Gerontology of the National Academy of Medical Sciences of Ukraine, Kyiv, Ukraine; ^3^ Department of Cytology, Bogomoletz Institute of Physiology, National Academy of Sciences of Ukraine, Kyiv, Ukraine, nas.gov.ua; ^4^ Department of Intensive Care and Resuscitation, Strazhesko National Scientific Center of the National Academy of Medical Sciences of Ukraine, Kyiv, Ukraine

**Keywords:** acute coronary syndrome, cellular senescence, COVID-19, cytokinesis-block micronucleus (CBMN) cytome assay, senescence-associated *β*-galactosidase (SA-*β*-gal), senescence-associated heterochromatin foci (SAHF)

## Abstract

The purpose of this study was to investigate cytological markers of cellular senescence—including senescence‐associated *β*‐galactosidase (SA‐*β*‐gal), senescence‐associated heterochromatin foci (SAHF), and parameters of the cytokinesis‐block micronucleus (CBMN) cytome assay—in peripheral blood samples from patients who had experienced an acute coronary syndrome accompanied by COVID‐19 (Group 2), compared with similar patients without COVID‐19 (Group 1). The relative proportion of SA‐*β*‐gal–positive leukocytes was approximately threefold higher in patients with a history of COVID‐19 compared with Group 1. Group 2 also demonstrated a significantly higher frequency of SAHF‐positive leukocytes. According to the CBMN assay, Group 2 showed a significantly higher relative content of binucleated leukocytes with micronuclei, as well as a higher frequency of apoptotic mononuclear cells. No significant differences were observed between the groups in the relative content of binucleated cells with nuclear buds or nucleoplasmic bridges, nor in the proportion of necrotic mononuclear cells. The cytokinesis‐block proliferation index was comparable between the groups. The findings of this retrospective study may indicate a detrimental long‐term effect of COVID‐19 on the manifestation of cellular senescence at the organismal level, and the evaluated markers may be informative for developing new prognostic strategies for this patient population.

## 1. Introduction

Cardiovascular diseases remain the leading cause of mortality worldwide. With organismal aging, the cardiovascular system undergoes physiological changes that increase its susceptibility to disorders such as atherosclerosis, hypertension, and heart failure. Patients with cardiovascular pathology who have also had COVID‐19 are known to represent an extremely high‐risk cohort prone to rapid disease progression and complications [1, 2]. However, patients who have experienced exacerbations of cardiac pathology or acute cardiovascular events are considered to be at high cardiovascular risk, particularly under stress‐associated conditions such as the pandemic period and wartime [3]. Understanding the fundamental mechanisms of aging at the cellular level in the context of cardiovascular disease is therefore crucial for developing effective preventive and therapeutic strategies.

To this end, various methodological approaches are increasingly employed to assess cellular senescence markers in biological samples, including measurements of telomere length, evaluation of proinflammatory processes (levels of C‐reactive protein and interleukin‐6), indicators of oxidative stress (malondialdehyde and other markers), as well as analysis of changes in the epigenetic landscape, particularly DNA methylation patterns [4, 5]. The assessment of senescence‐associated *β*‐galactosidase (SA‐*β*‐gal) activity is among the most widely used and validated methods for detecting senescent cells. Increased SA‐*β*‐gal activity reflects a dysfunctional cellular state and correlates with additional hallmarks of senescence, including impaired proliferative capacity, metabolic alterations, and the accumulation of DNA damage [6].

These pathogenic components of cellular senescence converge into the concept of the senescence‐associated secretory phenotype (SASP). Activation of the SASP is characterized by excessive secretion of proinflammatory chemokines and cytokines, including IL‐8, IL‐6, granulocyte–macrophage colony‐stimulating factor, chemotactic mediators of the GRO family, adhesion molecules such as ICAM‐1, and monocyte chemoattractants MCP1‐4 [7, 8]. Virus‐induced cellular senescence and the associated reprogramming of cellular functions are key determinants of the hyperinflammatory response observed in SARS‐CoV‐2 infection. Similar to other pathogens, SARS‐CoV‐2 can trigger senescence and amplify the secretion of SASP components—proinflammatory cytokines, proteases, complement activators, and procoagulant molecules—secreted by senescent cells. In older individuals, these processes are markedly amplified due to an increased baseline pool of already‐senescent cells, contributing to a more intense and dysregulated inflammatory response [9, 10].

Cellular senescence is a complex biological program induced by diverse stressors, including DNA damage, oncogenic activation, oxidative stress, metabolic dysfunction, and chronic inflammation [11]. It is accompanied by a stable arrest of the cell cycle and profound chromatin remodeling, including the formation of specific nuclear structures known as senescence‐associated heterochromatin foci (SAHF) [12]. SAHF play an important role in stabilizing the senescent cell phenotype and enforcing proliferative arrest through the repression of E2F‐dependent gene expression. These structures were first described in detail by Narita et al. [13] and later confirmed in other senescence models, predominantly in fibroblast cultures [14, 15].

Patients at high cardiovascular risk frequently exhibit elevated levels of cellular stress, oxidative damage, and systemic inflammation, all of which predispose to DNA damage and the accumulation of senescent cells in the bloodstream [16]. DNA damage manifested, among other features, as micronuclei represents a key trigger of senescence, as it reflects underlying genomic instability. Loss of genome stability due to physical or genotoxic insults can lead not only to premature aging but also to chronic inflammation and the development of conditions such as cancer, diabetes, neurodegenerative disorders, and cardiovascular diseases [17].

However, most classical studies of DNA damage associated with cellular senescence have been performed in vitro, whereas investigations of these phenomena in human blood cells in vivo remain limited. This is partly due to the fact that SAHF and micronuclei do not form uniformly across different cell types or in all models of senescence, as well as the technical challenges associated with their detection in routine clinical samples. Nevertheless, recent evidence indicates that senescent cells can accumulate under chronic inflammatory conditions and viral infections, including COVID‐19, contributing to so‐called “inflammaging” and the epigenetic reprogramming of the immune system [18, 19]. The spread of cellular senescence through SASP‐mediated paracrine signaling decouples tissue damage from the direct cytopathic effects of SARS‐CoV‐2 and may further enhance SASP secretion, thereby increasing the overall senescent cell burden [20].

Thus, contemporary evidence indicates that cellular senescence is closely linked to the development of cardiovascular diseases, and blood‐based senescence markers may serve as indicators of complication risk and clinical trajectory. Characterizing senescent cells in the peripheral blood of patients with cardiovascular pathology may provide new insights into the mechanisms of aging and the course of age‐associated disorders, particularly in the context of prior COVID‐19 and under the stress‐related conditions of the pandemic and wartime. Such knowledge may also support the development of promising therapeutic strategies, including novel pharmacological and bioengineered cell‐based interventions.

However, personalized criteria for assessing high cardiovascular risk using cellular senescence markers have not yet been established. Such criteria would enable a deeper understanding of the interplay between aging and cardiovascular disease and could improve clinical management approaches for these patients. Peripheral blood is an easily accessible biological material; therefore, methods that enable the detection of senescence markers in circulating cells have substantial potential clinical relevance.

The aim of this study was to compare cytological markers of cellular senescence in the blood of high‐risk cardiovascular patients who experienced acute cardiac events prior to the COVID‐19 pandemic and those with concomitant COVID‐19.

## 2. Materials and Methods

The study was conducted in full compliance with bioethical principles and the provisions of the World Medical Association′s Declaration of Helsinki (1964), “Ethical Principles for Medical Research Involving Human Subjects.” The study protocol was approved by the local Ethics Committee of the Strazhesko National Scientific Center (Protocol #1–23 dated September 17, 2024). All participants voluntarily provided written informed consent prior to enrollment in the study.

### 2.1. Participants and Study Design

This retrospective cohort study included high cardiovascular‐risk patients who had been treated for acute coronary syndrome (ACS) presenting as ST‐segment elevation myocardial infarction (STEMI) in the Department of Intensive Care and Resuscitation at the M. D. Strazhesko National Scientific Center Institute of Cardiology, Clinical and Regenerative Medicine (NAMS of Ukraine).

The first group (*n* = 30) consisted of the patients who experienced ACS before the COVID‐19 pandemic, during the period from January 01, 2019 to March 31, 2020. The mean age at the time of evaluation was 62.1 ± 10.3 years. The group included 27 men and 3 women, with mean ages of 61.3 ± 10.2 years and 69.3 ± 10.0 years, respectively.

The second group consisted of the patients who experienced ACS accompanied by documented acute COVID‐19, during the period from April 1, 2020 to February 24, 2022. The mean age at the time of evaluation was 61.7 ± 10.7 years. This group included 24 men and 6 women, with a mean age of 60.2 ± 10.0 years and 67.8 ± 12.1 years, respectively. The examination and blood sampling in the second group was performed 44–58 months after inpatient treatment (average—49 months).

The study population was identified by screening all patients who had been discharged from the department during the abovementioned periods and who survived for the following 50 months. Inclusion criteria at screening were ACS with ST segment elevation admitted to the hospital within the first 6 h after symptom onset for all, concomitant acute viral infection of SARS‐CoV‐2 for the 2nd group, age over 40 until 80 years. Exclusion criteria were as follows: diabetes, cancer, severe heart failure, uncontrolled hypertension, chronic inflammatory diseases, chronic kidney disease, and anemia.Medical history after hospital discharge until inclusion to the study included documented episodes of COVID‐19 infections and vaccinations (verified with the National Electronic Health system), recurrent myocardial infarctions, myocardial revascularization (stenting or coronary artery bypass grafting), and all cardiovascular hospitalizations. A detailed clinical and demographic description of the patient groups is presented in Table 1.

**Table 1:** Clinical characteristics of patients included in this study.

Parameter	Group 1 (*n* = 30)	Group 2 (*n* = 30)	*p*
Age, years, mean ± SD (range)	62.1 ± 10.3 (76–85)	61.7 ± 10.7 (39–80)	*p* > 0.05
Gender, *n* (%)			
Male	27 (90%)	24 (80%)	*p* > 0.05
Female	3 (10%)	6 (20%)	*p* > 0.05
Angina pectoris	15 (50%)	12 (40%)	*p* > 0.05
Heart failure (NYHA I‐II)	19 (63.3%)	12 (40%)	*p* > 0.05
Arterial hypertension	11 (36.7%)	11 (36.7%)	*p* > 0.05
PAD	2 (6.7%)	4 (13.3%)	*p* > 0.05
Current smokers	6 (20%)	6 (20%)	*p* > 0.05
Obesity (BMI ≥ 30 kg/m^2^)	8 (26.7%)	12 (40%)	*p* > 0.05
Postdischarge history	12 (40.0%)	17 (56.7%)	*p* > 0.05
Recurrent myocardial infarction	3 (10%)	1 (3.3%)	*p* > 0.05
Myocardial revascularization due to unstable angina	8 (26.7%)	7 (23.3%)	*p* > 0.05
Cardiovascular hospitalizations	12 (40%)	17 (56.7%)	*p* > 0.05
SARS‐CoV‐2 infection	16 (53.3%)	21 (72.4%)	*p* > 0.05
COVID‐19 vaccination	17 (56.7%)	25 (83.3%)	*p* ≤ 0.05
Systematic treatment with three or more CV drugs	30 (100%)	28 (93.3%)	*p* > 0.05

### 2.2. Blood Sample Collection

Fasting peripheral venous blood samples were obtained from the patients in the morning and transported to the laboratory at 18°C–23°C within 60 min. For the detection of cells expressing SA‐*β*‐gal and senescent cells with chromatin reorganization features, blood was collected into vacuum tubes containing K_2_EDTA (Vacutest Kima, Italy). For the micronucleus assay in the mononuclear leukocyte fraction, blood was collected into tubes containing Li‐He anticoagulant (Vacutest Kima, Italy).

### 2.3. Flow Cytometry

For flow cytometric analysis, 100 *μ*L of fresh blood was transferred into 5‐mL polystyrene tubes and incubated with fluorochrome‐conjugated monoclonal mouse antihuman antibodies BD Pharmingen APC‐Cy7 mouse antihuman CD45 (Cat. No. 557833, Becton Dickinson, United States) and 1.5 *μ*L of senescence dye (Senescence Assay kit, Cat. No. ab228562, Abcam, United States). Samples were incubated for 60 min at 37°C in a humidified 5% CO_2_ atmosphere using a Steri‐Cycle 371 CO_2_ incubator according to manufacturer instruction (Thermo Scientific, United States).

After incubation, 2 mL of 1 × BD Pharm Lyse solution (Cat. No. 555899, BD Pharmingen, United States) was added to each tube, gently mixed on a microcentrifuge vortex (Biosan, Latvia), and incubated for 5 min at room temperature for erythrocyte lysis. To remove residual antibodies and lysis solution, samples were centrifuged at 300 × g for 5 min in an IEC CL30 centrifuge (Thermo Scientific, United States). The supernatant was discarded, and the cell pellet was resuspended in 300 *μ*L of BD CellWash buffer (Cat. No. 349524, BD, United States). Immediately prior to analysis, the cell suspension was filtered through a 40‐*μ*m cell strainer (Falcon, United States), and the vital dye 7‐aminoactinomycin D (7‐AAD) (Cat. No. 559925, BD Pharmingen, United States) was added to discriminate dead cells.

Measurements were performed using a BD FACSAria cell sorter (Becton Dickinson, United States). For each sample, at least 300,000 CD45‐positive leukocytes were recorded for subsequent analysis. To control autofluorescence and set compensation for spectral overlap during multiparameter analysis, unstained control samples and single‐stained control samples for each fluorochrome were used. Preliminary gating to exclude erythrocytes based on size and morphology was performed manually using forward scatter (FSC) and side scatter (SSC) parameters, followed by the exclusion of potential aggregates based on signal height and width. 7‐AAD–positive nonviable cells were discriminated on the PerCP‐Cy5.5 channel (695/40 nm). To accurately identify leukocytes among viable cells, gating of the CD45‐positive target population was applied on the APC‐Cy7 channel (780/60 nm). The relative proportion of cells expressing SA‐*β*‐gal was determined on the FITC channel (530/30 nm) among viable CD45‐positive leukocytes. Graphical data were visualized as bar and dot‐plot histograms, and numerical results were processed using BD FACSDiva 6.1.2 software (Becton Dickinson, United States).

### 2.4. Analysis of SAHF

The number of cells containing heterochromatin foci in nuclei was determined as previously described [21]. For fluorescence microscopy, 5 *μ*L of blood was placed on a microscope slide and spread to prepare a thin smear. Air‐dried smears were fixed in freshly prepared 4% formaldehyde vapor at room temperature for 30 min. After fixation, the slides were mounted with Fluoromount‐G Mounting Medium (Cat. No. 00‐4959‐52, Invitrogen, United States) containing the fluorescent dye DAPI (4 ^′^,6‐diamidino‐2‐phenylindole) and covered with a coverslip.

Cytological preparations were examined using an IX71 fluorescence microscope (Olympus, Japan) with an excitation wavelength of *λ*_ex_ ≈358 nm and an emission wavelength of *λ*_em_ ≈461 nm. Images were captured using a DP20 digital camera (Olympus, Japan) and analyzed with QuickPHOTO MICRO 2.3 software (Olympus, Japan). For each sample, more than 100 cells were assessed, and nuclei containing clearly defined, bright heterochromatin regions were counted as SAHF‐positive, according to Oizumi et al. [22]. Scoring was conducted in a blinded manner with respect to group allocation.

The relative proportion of SAHF‐positive cells was calculated using the formula:

SAHF−positive cells,%=number of SAHF−positive cellstotal number of counted cells×100%.

### 2.5. Cytokinesis‐Block Micronucleus (CBMN) Cytome Assay

The micronuclei assay was performed on short‐term cultures of peripheral blood mononuclear cells (PBMCs) as previously described [23]. PBMCs were isolated using a density gradient with Lymphocytes Separation Media 1.077 g/mL (Cat. No. LSM‐A, Capricorn Scientific GmbH, Germany). Whole blood samples were diluted 1:2 with DPBS (Cat. No. PBS‐1A, Capricorn Scientific GmbH, Germany), carefully layered onto the Ficoll gradient, and centrifuged at 400 × g for 30 min.

After washing and counting with a Neubauer hemocytometer, 1 × 10^6^ isolated mononuclear cells were transferred into 5‐mL polystyrene culture tubes and cultured at 37°C with 5% CO_2_ in a humidified CO_2_ incubator (Steri‐Cycle 371, Thermo Scientific, United States). The complete culture medium consisted of RPMI‐1640 (Cat. No. RPMI‐XA, Capricorn Scientific GmbH, Germany) supplemented with 10% fetal bovine serum (Cat. No. FBS‐12B, Capricorn Scientific GmbH, Germany), 1% penicillin/streptomycin (Cat. No. P11‐010, PAA Laboratories GmbH, Germany), 1% L‐glutamine solution (Cat. No. M11‐006, PAA Laboratories GmbH, Germany), and 30‐*μ*g/mL phytohemagglutinin M (Cat. No. 11082132001, Roche Diagnostics GmbH, Germany).

At 44 h of culture, cytochalasin B (Cat. No. C6762, Sigma, United States) was added at a final concentration of 6 *μ*g/mL to block cytokinesis. After 72 h, the cell suspension was dropped from a height onto degreased microscope slides and fixed in methanol vapor for 10 min. The preparations were then stained with Giemsa solution (Cat. No. 48900‐500ML‐F, Sigma‐Aldrich GmbH, Germany) for 5–7 min. After air‐drying, the slides were examined under an IX71 microscope (Olympus, Japan), photographed with a DP20 digital camera (Olympus, Japan), and analyzed using QuickPHOTO MICRO 2.3 software (Olympus, Japan).

In each sample, 1000 binucleated cells were scored to determine the frequency of cells containing micronuclei, nucleoplasmic bridges, and nuclear buds, as well as apoptotic and necrotic cells. Additionally, the number of cells with 1, 2, 3, or 4 nuclei was counted to calculate the cytokinesis‐block proliferation index (CBPI) using the following formula:

CBPI=M1223344+M+M+MN.

M_1_–M_4_ represent the numbers of cells with 1–4 nuclei, respectively, and *N* is the total number of viable cells scored (excluding apoptotic and necrotic cells).

### 2.6. Statistical Analysis

Statistical analyses were performed using STATISTICA 10.0 software (StatSoft Inc., 2011, United States). Normality of continuous variables was assessed using the Shapiro–Wilk test. Variables that were not normally distributed were analyzed using the nonparametric Mann–Whitney *U* test. Data are presented as mean ± SD for normally distributed variables or median (interquartile range, IQR) for nonnormally distributed variables to reflect biological variability. A *p* value ≤ 0.05 was considered statistically significant, and ≤ 0.001 was considered highly statistically significant.

## 3. Results

Analysis of cytological markers of cellular senescence revealed differences in specific parameters between the comparison groups of patients who experienced ACS before the COVID‐19 pandemic and those with concomitant documented acute COVID‐19 (Table 2).

**Table 2:** Comparative characteristics of patient groups based on cytological markers of cellular senescence, median (IQR).

Parameter	Group 1 (*n* = 30)	Group 2 (*n* = 30)	*p*
Immunophenotyping of blood leukocytes			
SA‐*β*‐gal^+^ cells among leukocytes, %	0.012 (0.009–0.023)	0.042 (0.028–0.083)	*p* < 0.001
Analysis of chromatin reorganization in leukocyte nuclei			
Cells with SAHF among leukocytes, %	10.7 (8.9–13.5)	12.7 (10.7–15.0)	*p* < 0.05
CBMN assay of peripheral blood mononuclear leukocytes			
Cells with micronuclei (MNi) among binucleated leukocytes, %	0.3 (0.2–0.4)	0.9 (0.3–1.7)	*p* < 0.001
Cells with nuclear buds among binucleated leukocytes, %	0.1 (0.1–0.2)	0.0 (0.0–0.2)	*p* > 0.05
Cells with nucleoplasmic bridges among binucleated leukocytes, %	0.1 (0.0–0.2)	0.0 (0.0–0.1)	*p* > 0.05
Cells in apoptosis among mononuclear cells, %	1.1 (0.6–1.6)	2.8 (1.2–5.4)	*p* < 0.05
Cells in necrosis among mononuclear cells, %	0.4 (0.4–0.6)	0.0 (0.0–0.4)	*p* > 0.05
Cytokinesis‐block proliferation index (CBPI)	1.8 (1.7–1.9)	1.8 (1.7–1.8)	*p* > 0.05

*Note:* Group 1—patients who experienced ACS before the COVID‐19 pandemic; Group 2—patients who experienced ACS with concomitant documented COVID‐19.

Abbreviations: SA‐*β*‐gal, senescence‐associated *β*‐galactosidase; SAHF, senescence‐associated heterochromatin foci; CBMN, cytokinesis‐block micronucleus cytome assay.

Flow cytometry analysis of blood cells revealed a significantly higher proportion of SA‐*β*‐gal–positive leukocytes in Group 2 (0.042% [0.027–0.083]) compared with Group 1 (0.012% [0.008–0.023]; *p* < 0.001), representing an approximately threefold higher proportion (Figure 1). This typical lysosomal senescence marker reflects the cellular response to damaging endogenous or exogenous factors. Although the fluorescence signal intensity was low, the applied method enabled the detection of statistically significant differences between the comparison groups.

**Figure 1:**
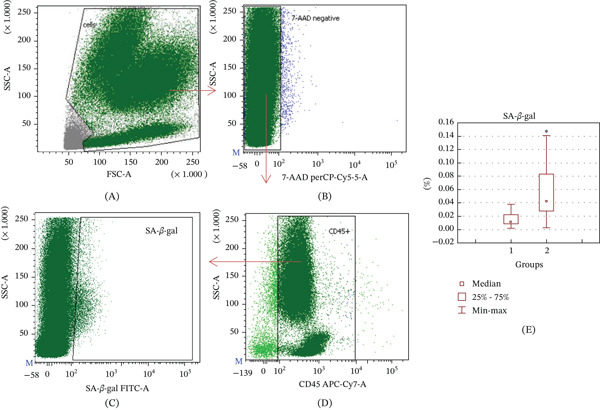
Representative dot‐plot histograms of human peripheral blood samples showing gating strategies for SA‐*β*‐gal expression analyzed by (A–D) flow cytometry, along with bar graphs of (E) the statistical analysis, ∗—*p* ≤ 0.001.

For the quantitative assessment of SAHF in patient blood cells, a standard protocol for preparing peripheral blood smears was used, followed by DAPI fluorescent staining to clearly visualize compact heterochromatin domains. After fixation and incubation with the dye, the smears were analyzed using a fluorescence microscope. In each sample, more than 100 leukocyte nuclei were evaluated and classified as SAHF‐negative or SAHF‐positive. SAHF foci were defined as sharply delineated, intensely stained regions of condensed heterochromatin against a uniformly stained nuclear background (Figure 2).

**Figure 2:**
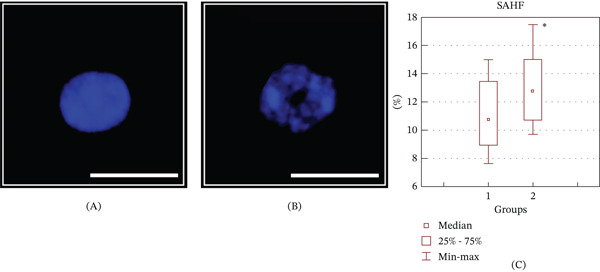
Representative micrographs of human blood leukocytes in blood smears with DAPI‐stained nuclei: (A) nucleus without SAHF; (B) nucleus with SAHF, along with (C) bar graphs of the statistical analysis, ∗—*p* ≤ 0.05. Fluorescence microscopy, scale bar = 10 *μ* *m*.

In Group 1, the median (IQR) percentage of leukocytes with SAHF was 10.7% (8.9–13.5). In Group 2, comprising patients who experienced acute cardiovascular events in combination with COVID‐19, the percentage of SAHF‐positive leukocytes was higher at 12.7% (10.7–15.0), and this difference was statistically significant (p <0.05). Thus, Group 2 was characterized by an increased level of nuclear chromatin reorganization, indicating a stronger cellular senescence phenotype. In addition, in Group 2, SAHF were not only more frequent than in Group 1 but were also often larger and more intensely stained, suggesting enhanced heterochromatin compaction (Figure 2).

Overall, these data suggest that a history of ACS with concomitant acute COVID‐19 may be associated with an increased proportion of peripheral blood cells exhibiting profound nuclear reorganization characteristic of the senescent phenotype.

We performed a micronucleus analysis of PBMCs stimulated to divide in vitro, followed by cytokinesis‐block to assess genomic instability as a consequence of DNA damage associated with senescence. The analysis included counting binucleated cells with micronuclei, nuclear buds, nuclear bridges, as well as necrotic and apoptotic cells Figure 3.

**Figure 3:**
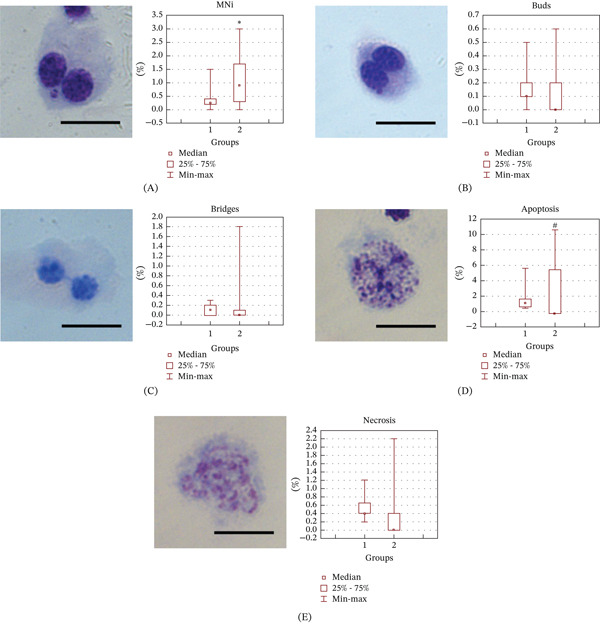
Representative micrographs of peripheral blood mononuclear cells analyzed by the CBMN assay along with corresponding bar graphs of group data,  ^∗^—*p* ≤ 0.001,  ^#^—*p* ≤ 0.05. (A) cell with micronucleus, (B) cell with nuclear bud, (C) cell with nucleoplasmic bridge, (D) apoptotic cell, and (E) necrotic cell. Giemsa staining, light microscopy, scale bar = 10 *μ* *m*.

Nuclear buds are defined as protrusions of the nucleus containing chromosomal fragments or amplified DNA segments. Nuclear bridges between daughter nuclei form during cell division due to defects in DNA repair, improper chromosome segregation, or incorrect joining of chromosome ends (e.g., resulting from fusions or chromosomal translocations). Additionally, the CBPI was calculated based on the distribution of cells with varying numbers of nuclei.

The assessment of genomic instability using the CBMN assay in short‐term cultures of PBMCs revealed a significantly higher proportion of micronucleated (MNi) binucleated leukocytes in Group 2 patients, as well as an increased proportion of apoptotic mononuclear cells, compared with Group 1 (*p* < 0.05). No significant differences were observed between the groups in the frequencies of binucleated cells with nuclear buds (Buds) or nucleoplasmic bridges (Bridges), nor in the proportion of necrotic mononuclear cells. The CBPI, reflecting the number of cell divisions following cytokinesis inhibition, was nearly identical in both groups (~1.8).

## 4. Discussion

In our study, we performed a comprehensive analysis of cytological markers of cellular senescence in peripheral blood samples from patients with high cardiovascular risk, taking into account the potential impact of prior COVID‐19 with ACS. Flow cytometry was used to determine the proportion of cells expressing SA‐*β*‐gal, immunofluorescent detection of SAHF was employed to assess nuclear chromatin reorganization, and a combined CBMN cytological analysis on short‐term cultured mononuclear cells in vitro was used to evaluate the consequences of genomic instability during cell division.

SA‐*β*‐gal activity is one of the most widely used indicators of cellular senescence, reflecting the increased production of this lysosomal enzyme in senescent cells. This marker represents functional changes in the lysosomal compartment that accompany the development of the SASP phenotype and often correlates with other features of senescence, such as metabolic remodeling, altered proliferative potential, and the secretion of proinflammatory mediators.

Typically, SA‐*β*‐gal is detected using cytochemical staining. However, this approach requires considerable time to count a statistically significant number of cells and is associated with high subjectivity in evaluation. Therefore, rapid quantification of a large number of cells in biological samples using flow cytometry offers several advantages, providing a more efficient and accurate analysis.

Kurz et al. [24] were among the first to propose the use of flow cytometry for assessing lysosomal SA‐*β*‐gal, enabling measurements in live cells and increasing assay sensitivity. They demonstrated that, regardless of pH changes, senescent cells exhibited SA‐*β*‐gal activity levels three‐ to six‐fold higher than those of young cells. Limitations of this approach include the need for lysosomal alkalization using chloroquine or bafilomycin A1 (Baf A1) and the adjustment of the intracellular environment to pH 6 with nigericin, which significantly complicates the procedure.

Noppe et al. [25] optimized the flow cytometry method described by previous researchers to better differentiate skin fibroblast populations according to SA‐*β*‐gal activity in young and very old subjects. They found that the modified method, using the pH modulator Baf A1—which is minimally toxic to fibroblasts—more accurately distinguished the degree of senescence across different cell populations. Under nonstressed conditions, fibroblasts from very old individuals exhibited higher SA‐*β*‐gal activity than those from young individuals. Under stress conditions, flow cytometry—but not the cytochemical method—detected significantly higher SA‐*β*‐gal activity in fibroblasts from very old individuals compared with young individuals.

Recently, Pangrazzi et al. [26] described another improved method using the SPiDER *β*‐gal substrate from a senescence detection kit (Dojindo), designed to enhance cell permeability and enable quantitative assessment of senescence by flow cytometry in both live and fixed cells. They applied this protocol to detect SA‐*β*‐gal in PBMCs isolated from healthy donors of different ages and demonstrated an age‐related progressive increase in SA‐*β*‐gal^+^ CD8^+^ T cells. According to results of Martínez‐Zamudio et al. [27], the relative proportion of cells with SA‐*β*‐gal activity is lowest (less than 5%) in cord blood, including T lymphocytes. With aging, an increasing fraction of T lymphocytes—particularly CD8^+^ T cells—develop high SA‐*β*‐gal activity (on average, 64%)

In our study, we used the Senescence Assay kit (Abcam, United States) on whole blood samples and demonstrated an increased proportion of SA‐*β*‐gal^+^ leukocytes within the overall leukocyte population in patients who had experienced acute cardiovascular events in the context of concomitant acute COVID‐19. Although the relative levels of SA‐*β*‐gal^+^ cells we identified are quite low, they may differ from results reported by other researchers using alternative detection approaches. Studies investigating senescent cells in whole blood samples remain relatively limited, and in most of those available to us, lysosomal pH modulators were used. However, their application is not mandatory; rather, it serves as a strategy to enhance signal sensitivity and contrast.

In the present study, senescent cells in peripheral blood were assessed in fresh, unfixed samples without the use of lysosomal modulators in order to avoid artificial enhancement of lysosomal activity. We recognize the potential limitations of detecting SA‐*β*‐gal under these conditions, which may result in lower sensitivity. Therefore, we assume that detection of SA‐*β*‐gal–positive cells using the Abcam kit without additional lysosomal pH modulators primarily reflects the presence of senescent cells expressing SA‐*β*‐gal at a basal level in native blood samples without additional processing or fixation. This approach prioritizes specificity over maximal sensitivity.

Unfortunately, we were unable to identify comparable studies using this specific detection kit in blood samples to directly compare our findings. We hope that further studies will become available to clarify this issue. It is also important to emphasize that our focus was primarily on comparative analysis between groups rather than on absolute values, using a combination of senescence markers. Importantly, the observed differences were consistent across independent senescence‐associated readouts, supporting the biological relevance of the findings.

One approach to assessing nuclear chromatin reorganization during senescence is the staining of blood cells with the fluorescent dye DAPI, which allows the identification of SAHF as compact, well‐defined, and brightly stained heterochromatin regions. Although DAPI is not a specific SAHF marker, it reliably visualizes their morphology, as confirmed by numerous studies in culture models [3]. Nevertheless, definitive attribution of DAPI‐dense foci to senescence‐associated heterochromatin typically requires validation by colocalization with established heterochromatin markers, including HP1 isoforms (e.g., HP1*γ*), macroH2A, and repressive histone modifications such as H3K9me3. Accordingly, SAHF data are interpreted in the present study as a supportive morphological indicator of senescence‐related nuclear reorganization rather than as a standalone or definitive marker. Our results show that high cardiovascular risk patients who had recovered from ACS with concomitant acute COVID‐19 exhibit higher levels of SAHF in blood cells compared with similar‐profile patients without COVID‐19. This finding aligns with current evidence that SARS‐CoV‐2 infection in addition to acute cardiovascular event can trigger a strong wave of cellular senescence via DNA damage, mitochondrial dysfunction, and chronic inflammation [18, 20]. Studies analyzing immune cells from COVID‐19 patients have reported activation of p53/p21–dependent pathways, increased repressive epigenetic marks, and the formation of heterochromatin domains, consistent with the nature of SAHF [28].

The method of assessing SAHF in blood smears, despite its simplicity, allows evaluation of profound nuclear architectural remodeling—a process that otherwise requires cultured cells or biopsy material. DAPI‐based SAHF analysis is inexpensive, rapid, and accessible, potentially serving as an additional indicator of systemic cellular senescence in clinical laboratories. When combined with other markers (*β*‐galactosidase, micronucleus assay), this approach enables a more comprehensive assessment of the epigenetic state of leukocytes.

Our findings also suggest that SAHF may serve as a useful biomarker for estimating the risk of complications in patients with cardiovascular diseases. It is well established that senescence of immune cells contributes to the development of chronic inflammation (“inflammaging”), which acts as a catalyst for atherogenesis, vascular dysfunction, and thrombosis [29, 30]. Senescence‐associated changes in the immune system are also linked to increased severity and mortality of COVID‐19 in elderly individuals [19, 31]. Therefore, an increased frequency of SAHF may serve as an indirect indicator of these pathological processes.

Chronic inflammation can also induce chromosomal damage, leading to the formation of micronuclei—recognized biomarkers of genomic damage and chromosomal instability associated with numerous diseases. A high frequency of micronucleus formation indicates defects in DNA repair, mitotic errors, or exposure to genotoxins, and is linked to an increased risk of cancer, infertility, developmental abnormalities, neurological disorders, and other pathologies [17, 32]. Importantly, micronuclei are not unique specific markers of cellular senescence. They may also arise in response to acute inflammation, oxidative stress, environmental or pharmacological exposures, and other genotoxic stimuli.

Oxidative stress, a common manifestation of systemic inflammation in cardiovascular diseases, is a well‐established driver of DNA damage and micronucleus formation. In turn, DNA contained within micronuclei can activate proinflammatory signaling pathways, notably the cGAS‐STING pathway, thereby contributing to the persistence of chronic inflammation and the progression of atherosclerotic lesions [33]. In endothelial cells, micronuclei are considered indicators of DNA damage at the vascular level, and their increased frequency has been associated with more severe ischemic heart disease [34].

In our study, the observed increase in micronuclei should therefore be interpreted as evidence of enhanced genomic damage and chromosomal instability. Furthermore, the lack of significant changes in nucleoplasmic bridges and nuclear buds indicates that the observed genomic damage does not reflect a generalized rise in chromosomal instability, but rather points to a more specific or limited spectrum of chromosomal alterations. Taken together, our CBMN findings support an association between the studied condition and elevated genomic damage, while not implying a causal relationship or attributing the effect solely to cellular senescence.

Additional exposure to SARS‐CoV‐2 may further exacerbate these detrimental effects. COVID‐19 is known to activate p53/p21 pathways and induce a range of epigenetic changes [20]. Thus, the analysis of DNA damage markers in the blood of COVID‐19 patients may reflect the systemic response to viral infection, the degree of cellular stress, and the risk of developing complications. Accordingly, the study of SAHF and micronuclei in peripheral blood cells acquires particular importance as a potential noninvasive biomarker of systemic cellular senescence.

The increased frequency of micronuclei in peripheral blood leukocytes observed among Group 2 patients may indicate DNA damage induced by SARS‐CoV‐2. This damage leads to errors during cell division, causing chromosomal fragments or whole chromosomes to remain outside the main nucleus during mitosis. These events result from mitotic apparatus disruption, double‐strand DNA breaks, and replication stress, whereas the resulting micronuclei can further initiate and amplify genomic instability and inflammatory responses [35]. Additionally, the use of antiviral drugs for COVID‐19 treatment may also contribute to DNA damage [36, 37].

Thus, the accumulation of micronuclei caused by chromosomal damage, together with the formation of heterochromatin foci, sustains the systemic inflammatory response and can trigger a self‐perpetuating cycle that plays a significant role in the progression of cardiovascular diseases. Establishing correlations between markers of chromosomal instability and the extent of atherosclerosis could help evaluate their relevance in the pathogenesis of endothelial dysfunction. Overall, the level of DNA damage, as assessed by the analysis of heterochromatin foci and micronuclei in peripheral blood cells, could be considered a potentially associated marker of disease severity and complication risk in patients with heart disease; however, its clinical relevance requires validation in further dedicated studies.

Although the lifespan of peripheral blood leukocytes is relatively short compared with the timeframe of our retrospective study, we hypothesize that the detrimental effects of COVID‐19 are systemic and long‐lasting. Specifically, these effects may involve the activation of senescence in bone marrow stem and progenitor cells responsible for the continuous renewal of the blood cell pool. If these cells acquire a senescent phenotype, pathological changes can be transmitted to all their progeny, leading to the persistence of cellular senescence markers long after the acute infection. This mechanism could partially explain the sustained elevation of senescence markers observed in our study even months after the infection and highlights the need for further research on the impact of COVID‐19 on the regenerative potential of the hematopoietic system.

The increased proportion of leukocytes expressing SA‐*β*‐gal, exhibiting SAHF, and forming micronuclei during cell division in patients who recovered from COVID‐19 may reflect an association with systemic, prolonged poststress cellular alterations consistent with senescence‐related features. In some patients, these changes appear to persist for months after infection. This observation aligns with the proposed concept of “post‐COVID cellular senescence” as a hypothesis‐generating framework that may contribute to the understanding of long‐term post‐COVID manifestations and a potential predisposition to thrombosis and endothelial dysfunction [38]. However, given the cross‐sectional design of the present study and the absence of longitudinal outcome data, these findings should be interpreted cautiously and not as evidence of a definitive pathogenic mechanism.

Thus, we have demonstrated that comprehensive assessment of cellular senescence markers in leukocytes represents a promising approach for monitoring systemic cellular senescence in patients with cardiovascular risk, and that prior COVID‐19 infection significantly amplifies these processes. Our findings complement previous reports showing that circulating blood cells can exhibit senescence features, particularly in patients with metabolic and cardiovascular diseases. Recently, considerable attention has focused on the immune system′s response to SARS‐CoV‐2 infection, the interplay between severe COVID‐19, immunosenescence, cellular senescence, vaccination, and the emerging challenge of Long‐COVID [39]. Specific senescence endotypes (“sendotypes”) have been identified as potential determinants of COVID‐19 severity and mortality [40]. These observations open new avenues for developing personalized prevention and treatment strategies aimed at preserving cardiovascular health during physiological aging.

We consider it advisable to further develop scientifically grounded approaches for the comprehensive assessment and prediction of cardiovascular complications in patients with high cardiovascular risk, using novel cellular senescence markers that have not previously been studied in a clinical‐prognostic context. Given the limited sample size and retrospective design, multivariable modeling was not feasible. Nevertheless, we sought to emphasize the pathogenetic relevance of identifying potentially associated and diagnostic markers of cellular senescence in patients following COVID‐19. Future studies with larger patient cohorts, extended follow‐up periods, and a broader panel of epigenetic markers will be necessary to clarify the underlying mechanisms and determine the clinical significance of the observed changes.

Overall, elucidating the fundamental mechanisms by which cellular senescence influences the course of cardiovascular pathology in patients who have experienced acute cardiac events could provide a basis for implementing pathogenetically informed approaches to risk assessment of cardiovascular complications. This, in turn, may enhance the effectiveness of personalized therapy and reduce disability and mortality rates in these patients.

## 5. Conclusions

In our retrospective study, we observed elevated levels of cellular senescence markers—SA‐*β*‐gal, SAHF, and CBMN—in peripheral blood samples from patients who experienced acute cardiac events in the context of concurrent acute COVID‐19 infection, compared with similar patients without COVID‐19. These findings may indicate a prolonged detrimental effect of COVID‐19 in addition to acute cardiovascular events on the manifestations of cellular senescence at the organismal level.

Further investigation into the mechanisms linking cellular senescence with the course of cardiovascular pathology—particularly in the context of prior COVID‐19 infection and stress factors associated with wartime conditions—may contribute to the development of more effective diagnostic, therapeutic, and prognostic strategies for these patients. This, in turn, could provide substantial social and economic benefits to the healthcare system in the face of similar challenges in the future.

## Author Contributions

V.K.: flow cytometry, conceptualization, and writing and original draft submission; A.U.: CBMN assay and writing (original draft); O.T.: SAHF analysis and writing (original draft); O.S.: patient recruitment, clinical observation, and blood sampling; O.P.: supervision and review and editing.

## Funding

The research project titled “Development of personalized criteria for assessing high cardiovascular risk based on markers of cellular senescence” was conducted with grant support from the National Research Foundation of Ukraine (2023.03/0048).

## Disclosure

All authors have read and approved the final manuscript.

## Conflicts of Interest

The authors declare no conflicts of interest.

## Data Availability

The datasets supporting the conclusions of this article are included within the article.
